# An mRNA Vaccine Targeting the C-Terminal Region of P1 Protein Induces an Immune Response and Protects Against *Mycoplasma pneumoniae*

**DOI:** 10.3390/ijms26136536

**Published:** 2025-07-07

**Authors:** Fenglian Zhang, Chengwei Li, Yanan Wu, Hongyun Chuan, Shaohui Song, Yun Xie, Qi Zhu, Qianqian Chen, Fei Tong, Runfang Zhang, Guangbo Yuan, Xiaoyan Wu, Jian Zhou, Guoyang Liao

**Affiliations:** Institute of Medical Biology, Chinese Academy of Medical Sciences and Peking Union Medical College, Kunming 650118, China; zhangfl05271021@163.com (F.Z.); s2023018020@pumc.edu.cn (C.L.); wuyanancx88@126.com (Y.W.); chuanhongyun@imbcams.com.cn (H.C.); shaohuisong@126.com (S.S.); xieyun@imbcams.com.cn (Y.X.); zhuqii@pumc.edu.cn (Q.Z.); b2023018009@pumc.edu.cn (Q.C.); b2024018008@student.pumc.edu.cn (F.T.); zrf_0625@163.com (R.Z.); guangboyuan666@163.com (G.Y.); wuxiaoyan@imbcams.com.cn (X.W.)

**Keywords:** *Mycoplasma pneumoniae*, mRNA vaccine, P1 protein, ATCC M129 strain, ST3 drug-resistant strain

## Abstract

*Mycoplasma pneumoniae*, a cell wall-deficient pathogen, primarily affects children and adolescents, causing *Mycoplasma pneumoniae pneumonia* (MPP). Following the relaxation of non-pharmaceutical interventions (NPIs) post COVID-19, there has been a global increase in MPP cases and macrolide-resistant strains. Vaccination against *M. pneumoniae* is being explored as a promising approach to reduce infections, limit antibiotic misuse, and prevent the emergence of drug-resistant variants. We developed an mRNA vaccine, mRNA-SP+P1, incorporating a eukaryotic signal peptide (tissue-type plasminogen activator signal peptide) fused to the C-terminal region of the P1 protein. Targeting amino acids 1288 to 1518 of the P1 protein, the vaccine was administered intramuscularly to BALB/c mice in a three-dose regimen. To evaluate immunogenicity, we quantified anti-P1 IgG antibody titers using enzyme-linked immunosorbent assays (ELISAs) and assessed cellular immune responses by analyzing effector memory T cell populations using flow cytometry. We also tested the functional activity of vaccine-induced sera for their ability to inhibit adhesion of the ATCC M129 strain to KMB17 cells. The vaccine’s protective efficacy was assessed against the ATCC M129 strain and its cross-protection against the ST3-resistant strain. Transcriptomic analysis was conducted to investigate gene expression changes in peripheral blood, aiming to uncover mechanisms of immune modulation. The mRNA-SP+P1 vaccine induces P1 protein-specific IgG antibodies and an effector memory T-cell response in BALB/c mice. Adhesion inhibition assays demonstrated that serum from vaccinated mice attenuatesthe adhesion ability of ATCC M129 to KMB17 cells. Furthermore, three doses of the vaccine confer significant and long-lasting, though partial, protection against the ATCC M129 strain and partial cross-protection against the ST3 drug-resistant strain. Transcriptome analysis revealed significant gene expression changes in peripheral blood, confirming the vaccine’s capacity to elicit an immune response from the molecular level. Our results indicate that the mRNA-SP+P1 vaccine appears to be an effective vaccine candidate against the prevalence of *Mycoplasma pneumoniae*.

## 1. Introduction

*Mycoplasma pneumoniae* (*M. pneumoniae*), a cell wall-deficient atypical pathogen, primarily affects children and adolescents, causing *Mycoplasma pneumoniae* pneumonia (MPP) [1,2]. MPP is characterized by symptoms such as fever, cough, headache, and sore throat [3]. Although MPP often resolves spontaneously, it can occasionally progress to severe pneumonia [4,5]. Recent studies have shown that up to 25% of patients with *M. pneumoniae* infection may present with extrapulmonary manifestations, such as aseptic meningitis and myocarditis [6,7]. Globally, *M. pneumoniae* epidemics exhibit a periodicity, recurring every 3–7 years and lasting 1–2 years [8]. Following the relaxation of non-pharmaceutical interventions (NPIs) during the COVID-19 pandemic, there has been a notable resurgence in MPP cases worldwide [9,10,11]. Before the pandemic, the global incidence of MPP was 8.61%. This rate dropped to 0.7% during the strict enforcement of NPIs from 2020 to 2022. However, from April to October 2023, the infection rate among children rose to an average of 4.12%, with some regions surpassing pre-pandemic levels [12,13].

Macrolides, tetracyclines, and fluoroquinolones effectively treat *M. pneumoniae* infections [14,15]. However, rising macrolide resistance, notably in East Asia (mainland China 79.5%, Japan 47.3%, and Taiwan Province of China 32.4%) [16], presents a significant challenge. Infections by macrolide-resistant *Mycoplasma pneumoniae* (MRMP) result in prolonged fevers and increased treatment costs [17,18]. Although tetracyclines and fluoroquinolones are viable alternatives for MRMP treatment [19,20], their use in children is limited due to potential adverse effects on bone and cartilage development [21]. Despite low current resistance rates to these drugs, their extensive use could accelerate resistance development in *M. pneumoniae* [22]. Consequently, *M. pneumoniae* vaccination is a promising strategy to reduce infection rates, limit antibiotic use, and prevent resistant strain emergence.

The development of *M. pneumoniae* vaccines remains exploratory, with no approved products yet available. Historically, the first candidate, an inactivated vaccine from the 1960s, faced setbacks due to vaccine-enhanced disease (VED) in some participants [23,24]. Recent research identifies the lipid moieties of *M. pneumoniae* lipoproteins as the causative factor of VED. Removing these lipid moieties effectively prevents VED and reduces bacterial loads after infection, laying a critical foundation for safer vaccine development [25]. Advances in mRNA vaccine technology offer promising avenues for *M. pneumoniae* vaccines by encoding specific antigens rather than whole pathogens, thus circumventing the VED risks of traditional vaccines while preserving immunogenicity. Given the demonstrated safety and adaptability of mRNA platforms, as seen in COVID-19 vaccines, this approach holds potential for tackling the epidemiological and drug resistance challenges of *M. pneumoniae* [26,27,28,29].

The development of *M. pneumoniae* mRNA vaccines focuses on identifying antigen targets that effectively trigger specific immune responses. Research indicates that the core pathogenic mechanism of *M. pneumoniae* involves its P1 adhesin, which facilitates colonization and subsequent damage by binding specifically to receptors on respiratory epithelial cell surfaces [30]. Polyclonal antibodies against the C-terminal domain of the P1 protein (Lys1376-Asp1521 and Ala140-Asp1521) significantly inhibit this adhesion [31]. Subsequent research indicates that this domain is relatively conserved genetically [31]. Therefore, this study aims to construct an mRNA vaccine encoding the C-terminal of the P1 protein (amino acids 1288–1518), evaluate the induced humoral and cellular immune responses, and assess its cross-protective effect against the standard strain ATCC M129 and the drug-resistant strain ST3, offering a novel strategy for *M. pneumoniae* vaccine development.

## 2. Results

### 2.1. In Vitro Synthesis and Characterization of mRNA-SP+P1

**Figure 1:**
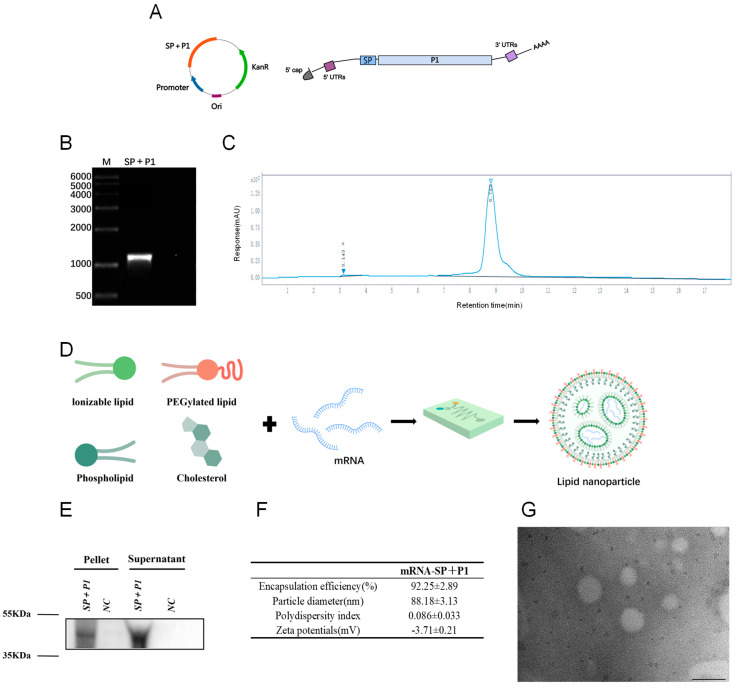
Design and encapsulation of mRNA-SP+P1. (**A**) Diagram of the SP+P1 mRNA construct, illustrating a fusion protein composed of a tissue-type plasminogen activator signal peptide (SP) linked to the C-terminal region (amino acids 1288–1518) of the P1 protein. (**B**) Formaldehyde-denatured agarose gel electrophoresis assessed the integrity of SP+P1. (**C**) High-performance liquid chromatography evaluated the purity of SP+P1. (**D**) Preparation of mRNA-SP+P1 involved mixing mRNA in an acidic aqueous solution, injecting organic phase lipids, and extruding the mixture through a microfluidic chip. (**E**) Expression of the C-terminal P1 protein region (1288aa–1518aa) was achieved in HEK293T cells transfected with SP+P1 using Lipofectamine 3000 for 48 h. (**F**) Physicochemical properties of mRNA-SP+P1 are presented as mean ± SD. (**G**) A representative TEM image illustrates the morphology of mRNA-SP+P1, with a scale bar of 100 nm.

The C-terminal region of the P1 protein (1288aa–1518aa) was selected as the antigenic target for mRNA synthesis. The mRNA (SP+P1) was constructed by integrating the coding sequence with UTRs and a poly(A) tail, then, it was cloned into pUC57 (Figure 1A). High-purity mRNA for vaccine purposes was obtained using in vitro transcription from linearized DNA (Figure 1B,C). Western blot analysis confirmed the expression and secretion of a 40 kDa P1 fragment, indicating successful translation (Figure 1E). The mRNA was encapsulated in LNPs (mRNA-SP+P1, Figure 1D), with an average size of 88.18 nm, a zeta potential of −3.71 mV, and an encapsulation efficiency of 92.25% (Figure 1F). TEM showed spherical nanoparticles, and no obvious particle aggregation was observed.

### 2.2. The Prophylactic Administration of mRNA-SP+P1 Induces Potent Humoral Immunity and Effector Memory T Cell Responses in Mice

**Figure 2:**
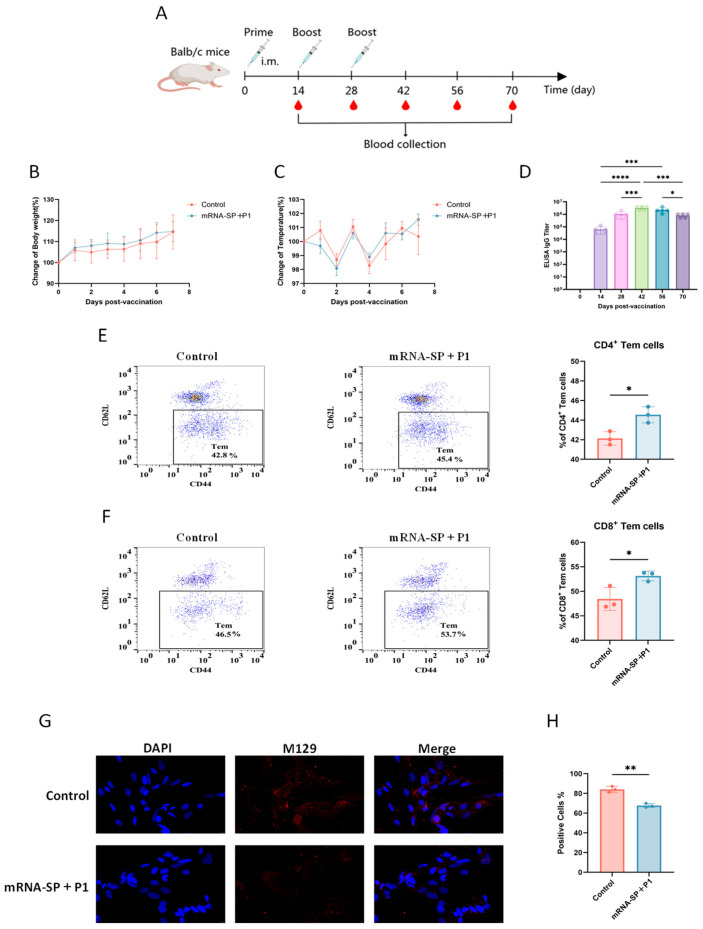
Humoral and effector memory T cell responses in mRNA-SP+P1-accinated mice. Female BALB/c mice received 20 μg of the mRNA-SP+P1 vaccine or served as controls (n = 5). Immunizations were administered intramuscularly on days 0, 14, and 28. Serum samples were collected on days 14, 28, 42, 56, and 70 post-initial immunization. (**A**) Immunization and sample collection timeline. (**B**,**C**) Body weight and temperature were monitored for seven days post-first immunization (n = 5). (**D**) The IgG antibody titers of P1 protein in the immunized group were evaluated by enzyme-linked immunosorbent assay (ELISA) (n = 5). Data are presented as mean ± SD. Statistical significance was determined using repeated measures ANOVA (n.s., not significant; * *p* < 0.05, ** *p* < 0.01, *** *p* < 0.001, **** *p* < 0.0001). (**E**,**F**) P1 protein-specific CD4^+^ and CD8^+^ effector memory T cells in the spleen were analyzed using flow cytometry (n = 3). (**G**,**H**) The inhibitory effect of serum from vaccinated mice on ACTT M129 adhesion to KMB17 cells was evaluated (n = 3). Positive cells were identified by specific fluorescent markers for *M. pneumoniae* on KMB17 cell surfaces, while negative cells lacked these markers. The data in (**E**,**F**,**H**) are presented as mean ± SD. Statistical significance was determined using an unpaired *t*-test (n.s., not significant; * *p* < 0.05, ** *p* < 0.01, *** *p* < 0.001).

The experimental group of mice received three intramuscular injections of mRNA-SP+P1 at 14-day intervals, whereas the control group was given mRNA-free LNP (Figure 2A). Over a 7-day post-immunization observation period, there were no significant differences in body temperature or weight between the groups, preliminarily indicating the vaccine’s safety (Figure 2B,C).

With successive immunizations, anti-P1 protein IgG antibody titers progressively rose: from 1 × 10^4^–10^5^ after the initial dose, to approximately 1 × 10^6^ post-second dose, and 1 × 10^6^–10^7^ after the third dose. However, by days 42 to 70, titers declined to 1 × 10^5^–10^6^ (Figure 2D). These results indicate that the mRNA-SP+P1 vaccine can activate the humoral immune response.

Vaccination stimulates an immune response, leading some T cells to differentiate into CD44^+^CD62L^−^ effector memory T cells (Tems), which rapidly activate upon pathogen re-exposure, eliciting a strong secondary immune response [32]. CD8^+^ Tems directly eliminate infected cells by secreting perforin and granzymes [33], while CD4^+^ Tems limit pathogen spread by releasing cytokines such as IFN-γ and TNF-α, and enhance the immune response by supporting B cells and CD8^+^ T cells responses [34]. Thus, measuring CD4^+^ Tem and CD8^+^ Tem levels post-vaccination can assess the cellular immune response and predict the vaccine’s long-term efficacy. We used flow cytometry to assess the mRNA-SP+P1 vaccine’s ability to generate P1 protein-specific Tems in mouse spleens. Results indicated significantly higher proportions of CD4^+^ Tem and CD8^+^ Tems in vaccinated mice compared to controls (Figure 2E,F), confirming the vaccine’s effectiveness in inducing effector memory T cells.

In summary, the mRNA-SP+P1 vaccine successfully elicited good humoral and memory T cell responses in mice.

### 2.3. The Serum of Mice Immunized with mRNA-SP+P1 Can Attenuate the Adhesion Ability of ATCC M129 to KMB17 Cells

The P1 protein of *M. pneumoniae*, a critical virulence factor located on the pathogen’s attachment organelle, is essential for infection. Antibodies against the P1 protein have been shown to effectively disrupt this adhesion [35] In our study, we performed an adhesion inhibition assay to evaluate the effect of immunized mouse sera on the adhesion of the ATCC M129 strain to KMB17 cells. Results indicated a significant reduction in *M. pneumoniae* adhesion to KMB17 cells when the bacterial suspension was co-incubated with mouse sera at 37 °C for 1 h (Figure 2G,H).

### 2.4. mRNA-SP+P1 Immunization Mitigates Infection Caused by ATCC M129 Challenge

**Figure 3:**
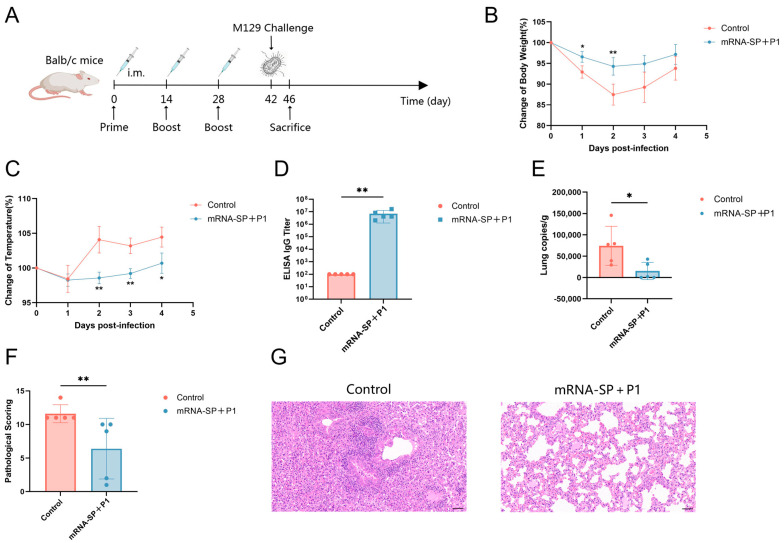
Efficacy of mRNA-SP+P1 in protecting mice against ATCC M129 challenge. (**A**) Immunization, challenge, and sample collection timeline. (**B**,**C**) Percentage changes in body weight and temperature post-infection (n = 5). (**D**) IgG antibody titers specific to *M. pneumoniae* antigen P1 post-infection (n = 5). (**E**) Lung *M. pneumoniae* load post-infection (n = 5). (**F**) Lung pathology scores post-infection (n = 5). (**G**) Lung histology with H&E staining post-infection; scale bar = 50 µm. Data are presented as mean ± SD. Statistical significance was determined using two-way ANOVA (**B**,**C**), Mann-Whitney test (**D**,**F**), or unpaired *t*-test (**E**) (n.s., not significant; * *p* < 0.05, ** *p* < 0.01, *** *p* < 0.001).

BALB/c mice received intramuscular injections of mRNA-SP+P1 on days 0, 14, and 28, followed by an intranasal challenge with ATCC M129 on day 42. Body temperature and weight were monitored daily post-infection. On the 4th day post-infection, mice were euthanized, and lung tissues and serum were collected (Figure 3A). Immunized mice exhibited less weight loss (Figure 3B), smaller temperature fluctuations (Figure 3C), higher anti-P1 IgG titers (Figure 3D), and reduced *M. pneumoniae* lung loads (Figure 3E) compared to controls. Histopathological analysis showed that lung damage in vaccinated mice was significantly reduced compared to the control group (Figure 3F,G). In the control group, mice exhibited severe alveolar damage, with extensive infiltration of granulocytes and lymphocytes, and necrotic cell debris. Conversely, these lesions were markedly less severe in the vaccinated group. Although the mRNA-SP+P1 vaccine did not completely prevent ATCC M129 infection, it significantly mitigated infection severity, lung *M. pneumoniae* loads, and pulmonary inflammation, demonstrating a protective effect.

### 2.5. mRNA-SP+P1 Immunization Mitigates Infection Caused by ATCC M129 Challenge on Day 62 After Booster Immunization

**Figure 4:**
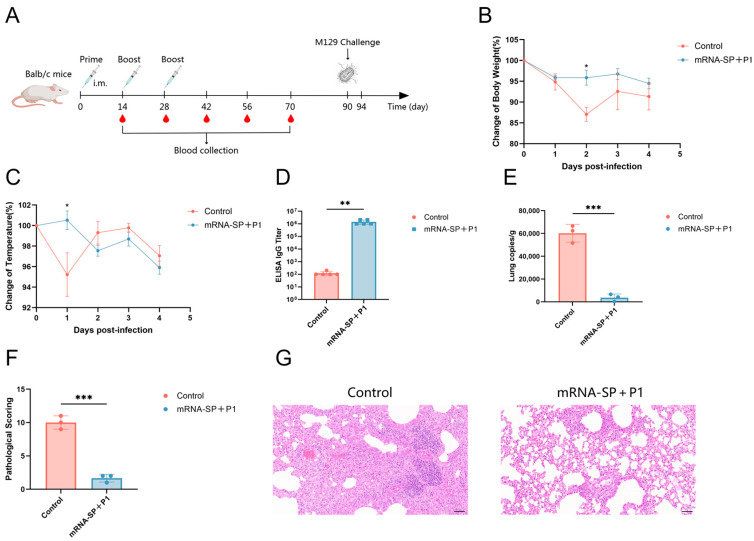
The protective effect of mRNA-SP+P1 against ATCC M129 in mice, as assessed 62 days post-booster immunization. (**A**) Immunization, challenge, and sample collection timeline. (**B**,**C**) Percentage changes in body weight and temperature post-infection (n = 5). (**D**) Titers of *M. pneumoniae*-specific P1 IgG antibodies post-infection (n = 5). (**E**) Lung *M. pneumoniae* load post-infection (n = 3). (**F**) Lung pathology scores post-infection (n = 3). (**G**) H&E-stained lung sections post-infection (n = 3), scale bar = 50 µm. Data are presented as mean ± SD. Statistical significance was determined using two-way ANOVA (**B**,**C**), Mann-Whitney test (**D**), or unpaired *t*-test (**E**,**F**) (n.s., not significant; * *p* < 0.05, ** *p* < 0.01, *** *p* < 0.001).

BALB/c mice were immunized with mRNA-SP+P1 on days 0, 14, and 28, followed by an intranasal challenge with ATCC M129 on day 90 (Figure 4A). Compared to controls, immunized mice experienced less body weight loss (Figure 4B), milder hypothermia (Figure 4C), elevated IgG titers against P1 protein (Figure 4D), and reduced *M. pneumoniae* lung load (Figure 4E). Histopathological analysis showed severe inflammation in controls, whereas vaccinated mice exhibited milder lung damage and near-normal lung structure (Figure 4F,G). Thus, on the 62nd day post-booster immunization, the mRNA-SP+P1 vaccine effectively mitigated weight and temperature changes and lung damage from ATCC M129 infection, significantly reducing *M. pneumoniae* lung load and demonstrating prolonged protective efficacy.

### 2.6. mRNA-SP+P1 Immunization Mitigates Infection Caused by the ST3-Resistant Strain of Mycoplasma pneumoniae

**Figure 5:**
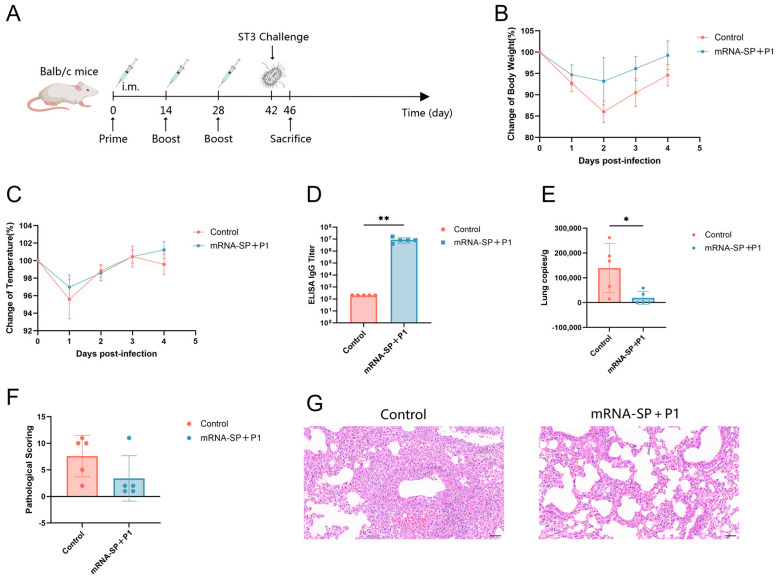
Efficacy of mRNA-SP+P1 in protecting mice against a challenge with an ST3-resistant strain on day 42 post-primary immunization. (**A**) Immunization, challenge, and sample collection timeline. (**B**,**C**) Percentage changes in body weight and temperature post-infection (n = 5). (**D**) IgG antibody titers specific to *M. pneumoniae* antigen P1 post-infection (n = 5). (**E**) *M. pneumoniae* load in lung tissue post-infection (n = 5). (**F**) Lung pathological scores post-infection (n = 5). (**G**) Lung histopathology with H&E staining post-infection (n = 5). Scale bar = 50 µm. Data are presented as mean ± SD. Statistical significance was assessed using two-way ANOVA (**B**,**C**) or Mann-Whitney test for non-parametric data (**D**,**F**) or unpaired *t*-test (**E**) (n.s., not significant; * *p* < 0.05, ** *p* < 0.01, *** *p* < 0.001).

To assess the efficacy of the mRNA-SP+P1 vaccine against drug-resistant *M. pneumoniae* in mice, 64 clinical isolates were cultured, with seven highly resistant strains selected for whole-genome sequencing to determine their multi-locus sequence typing (MLST) and antimicrobial resistance profiles. The ST3 drug-resistant strain (Table 1), prevalent in Asia (China 69.23%, Korea 74.7%) [36,37,38], was chosen as the infectious agent (Figure 5A). Mice in the immunization group exhibited less weight loss, stable body temperature (Figure 5B,C), higher anti-P1 protein IgG antibody titers, and reduced *M. pneumoniae* lung loads compared to controls (Figure 5D,E). While the pathological scores of lung tissues showed no statistical difference between the two mouse groups, the immunized group exhibited milder lung lesions and better-preserved tissue structure (Figure 5F,G). These findings suggest that the mRNA-SP+P1 vaccine offers partial cross-protection against infection by ST3 drug-resistant strains.

### 2.7. mRNA-SP+P1 Activates Immune System Responses

**Figure 6:**
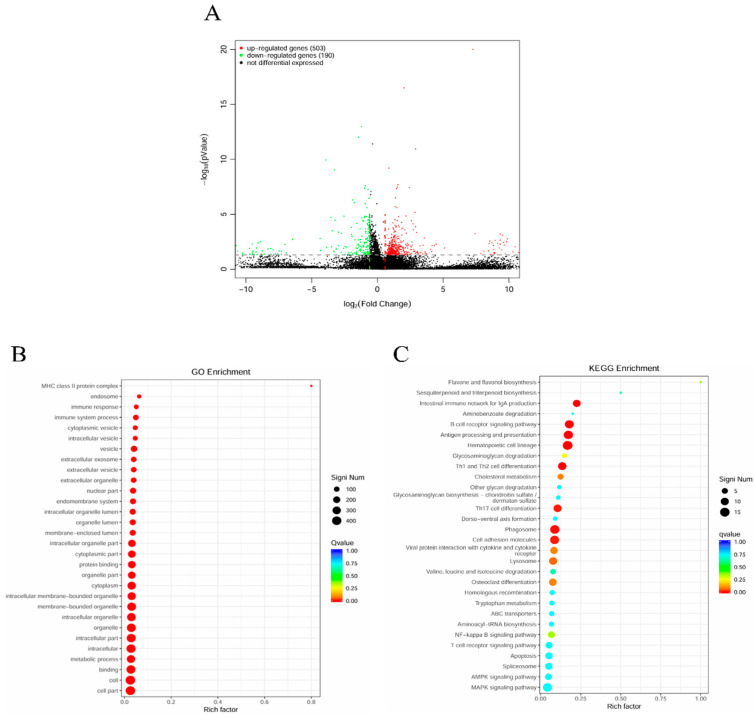
Transcriptome analysis of whole blood stimulated by mRNA-SP+P1. (**A**) Volcano plot illustrating differentially expressed genes, with upregulated genes in red and downregulated genes in green. (**B**) Gene Ontology (GO) enrichment analysis of upregulated genes, where the vertical axis denotes the GO term and the horizontal axis indicates the Rich factor. Dot size represents the number of genes per GO term, and dot color signifies Q value ranges. (**C**) Kyoto Encyclopedia of Genes and Genomes (KEGG) enrichment analysis of upregulated genes, with the vertical axis displaying significantly enriched pathways and the horizontal axis showing the Rich factor. Dot color reflects q value ranges.

To elucidate the molecular mechanisms driving the immune response elicited by the mRNA-SP+P1 vaccine, transcriptome sequencing was conducted on whole blood samples from both vaccinated and control mice. Differentially expressed gene (DEG) analysis identified 693 genes with significant expression differences between the groups. Of these, 503 genes were up-regulated and 190 were down-regulated in the vaccinated group relative to the control, suggesting a predominance of gene up-regulation (Figure 6A).

Gene Ontology (GO) enrichment analysis revealed that these upregulated differentially expressed genes were predominantly associated with the MHC class II protein complex, immune response, and immune system processes (Figure 6B). Kyoto Encyclopedia of Genes and Genomes (KEGG) analysis demonstrated significant enrichment of upregulated genes mainly in four immune-related pathways: the intestinal immune network for IgA production, B cell receptor signaling, antigen processing and presentation, and Th1 and Th2 cell differentiation (Figure 6C).

Transcriptomic analysis revealed that the mRNA-SP+P1 vaccine significantly alters gene expression in peripheral blood, demonstrating its capacity to activate the immune response at the molecular level.

## 3. Discussion

Following the relaxation of non-pharmaceutical interventions (NPIs), *M. pneumoniae* infections have resurged, complicating treatment due to its resistance to conventional antibiotics [39,40]. Vaccination emerges as a crucial strategy to address drug resistance by reducing infections and subsequent antibiotic use. In this study, we developed an mRNA vaccine, mRNA-SP+P1, targeting the C-terminal region of the P1 protein (1288aa–1518aa) using mRNA technology. Administered in three intramuscular doses to BALB/c mice, the vaccine elicited a humoral immune response and an effector memory T cell proliferation, providing significant and long-lasting, though partial, protection against the ATCC M129 strain and partial cross-protection against the drug-resistant ST3 strain. This approach offers a promising strategy for combating *M. pneumoniae* infections.

Designing an mRNA vaccine targeting the C-terminus (1288aa–1518aa) of *M. pneumoniae* P1 necessitates addressing differences in prokaryotic and eukaryotic expression systems. The P1 gene includes UGA codons, which encode tryptophan in prokaryotes but act as stop codons in eukaryotes [41], risking premature termination without codon optimization. Moreover, protein secretion pathways differ. Specifically, prokaryotes use the Sec/Tat pathway [42,43], while eukaryotes employ the SRP/Sec61 pathway [44]. Retaining the prokaryotic signal peptide could result in cytoplasmic retention and degradation of the P1 C-terminus, impairing antigen presentation. To address this, we employed a dual strategy: codon optimization for eukaryotic expression and substitution of the prokaryotic signal peptide with a eukaryotic secretion signal. Transfecting optimized mRNA into HEK293T cells demonstrated efficient target protein expression in both lysates and supernatants (Figure 1E).

The mRNA-SP+P1 vaccine, developed utilizing mRNA technology, demonstrates favorable immune protection in the BALB/c mouse model. This vaccine induces high levels of anti-P1 protein IgG antibodies (Figure 2D) and significantly expands effector memory T cells (Figure 2E,F). Even 62 days after booster immunization, it still mitigated infection caused by the ATCC M129 strain (Figure 4).

These results indicate that the protective efficacy may be jointly driven by humoral immunity and cellular immunity. Regarding humoral immunity, vaccine-induced specific antibodies are crucial in targeting the P1 protein-mediated adhesion process. Adhesion inhibition assays show that immune serum significantly reduces *M. pneumoniae* adhesion to KMB17 cells (Figure 2G,H), consistent with previous studies on the essential role of the P1 protein as an adhesin [45,46]. Simultaneously, at the cellular immunity level, effector memory T cells can rapidly proliferate and differentiate into effector T cells, initiating a secondary immune response against pathogens [47,48]. The dual protective mechanism of “antibody and memory cells” is vital in addressing cyclical *M. pneumoniae* outbreaks. Persistent antibody levels lower the infection threshold in susceptible populations, and the rapid response of effector memory T cells limits pathogen transmission, significantly aiding in the establishment of lasting herd immunity.

The prevalence of the drug-resistant *M. pneumoniae* ST3 strain in Asia led us to evaluate the mRNA-SP+P1 vaccine’s efficacy against this strain [36,37,38]. Our findings revealed that the vaccine conferred partial protection against the ST3 strain harboring resistance loci to macrolides, tetracyclines, and fluoroquinolones (Table 1) (Figure 5). This protective effect is likely attributed to two main factors. First, the amino acid sequence of the C-terminal region of the P1 protein, a crucial domain for adhesion function, remains relatively conserved in the ST3-resistant strain. This conservation enables vaccine-induced specific antibodies to impede the initial infection of the ST3-resistant strain by obstructing the interaction between the adhesin and host receptor. Second, the polyclonal antibodies generated by the mRNA-SP+P1 vaccine target multiple epitopes, mitigating the risk of immune evasion resulting from mutations in individual epitopes. While the vaccine did not significantly alleviate lung pathological damage induced by the ST3 drug-resistant strain (Figure 5F,G), it did reduce the persistent colonization of the strain in the host by decreasing lung *M. pneumoniae* load (Figure 5E). This presents an intervention approach for interrupting the infection cycle amid epidemics of drug-resistant strains.

Our research highlights the potential of the mRNA-SP+P1 vaccine against *M. pneumoniae* infection, yet several challenges remain for clinical translation. First, while the vaccine demonstrates partial protection against the ATCC M129 strain and the ST3 drug-resistant strain, its broad-spectrum efficacy requires further validation. This should include assessing cross-protection against other prevalent strains, such as ST7, ST14, and ST33. Second, this study assessed only one dose (20 μg) and one administration route (intramuscular injection). While this dose effectively induces humoral and cellular immune responses, conducting a dose-escalation study (e.g., 5–50 μg) remains essential to identify the minimum effective dose and minimize potential adverse reactions. Similarly, evaluating the immune responses and protective effects of other administration routes (such as intranasal administration, subcutaneous injection, etc.) is crucial for improving the vaccination schedule. Additionally, given the multifactorial pathogenesis of *M. pneumoniae*, developing a multivalent mRNA vaccine targeting P1 and other virulence factors, such as the CARDS toxin, could yield synergistic protective effects by disrupting the dual adhesion-damage pathway. Lastly, non-human primate studies are crucial to evaluate the vaccine’s efficacy in a model that closely mimics the human immune system.

## 4. Materials and Methods

### 4.1. Ethics Statement

All animal experiments received approval from the Institutional Animal Care and Use Committee of the Institute of Medical Biology, Chinese Academy of Medical Sciences (ethical approval number DWSP202110 005). The studies adhered strictly to Chinese regulations on experimental animals and complied with the standards for environmental and housing conditions.

### 4.2. Cells and Mycoplasma pneumoniae

HEK293T cells were cultured in Dulbecco’s modified Eagle medium (Thermo Fisher, Waltham, MA, USA) with 10% fetal bovine serum (Thermo Fisher, Waltham, MA, USA) and 100 U/mL penicillin streptomycin (Thermo Fisher, Waltham, MA, USA). KMB17 cells were maintained in DMEM (Thermo Fisher, Waltham, MA, USA) containing 7% FBS (Thermo Fisher, Waltham, MA, USA). Cultures were incubated at 37 °C in a humidified 5% CO_2_ atmosphere. *M. pneumoniae* was propagated in PPLO liquid medium and quantified using color-changing units (CCU), indicated by a medium color shift from red to yellowish orange.

### 4.3. Synthesis and Characterization of Mycoplasma pneumoniae mRNA

The signal peptide sequence of tissue-type plasminogen activator was fused to the codon-optimized C-terminal sequence (1288aa to 1518aa) of the P1 protein to create a “SP+P1” chimeric gene, subsequently cloned into an mRNA production plasmid (Sangon, Shanghai, China). The plasmid was transformed into DH5-α competent cells following the manufacturer’s protocol (Vazyme, Nanjing, China). Post-extraction, the plasmid was digested with BspQI (Vazyme, Nanjing, China) to produce a linear DNA template, then purified with DNA magnetic beads (Vazyme, Nanjing, China). In vitro transcription was conducted using T7 RNA polymerase (Vazyme, Nanjing, China), CleanCap (Syngenbio, Nanjing, China), and dNTPs, replacing UTP with N1-methylpseudouridine (m1ψ) (Syngenbio, Nanjing, China) to synthesize mRNA. The mRNA was subsequently purified using RNA magnetic beads (Vazyme, Nanjing, China). An ultraviolet spectrophotometer quantified mRNA concentration, while formaldehyde-denaturing agarose gel electrophoresis and high-performance liquid chromatography assessed mRNA integrity and purity, respectively.

HEK293T cells were transfected with 2.5 μg of mRNA using Lipofectamine 3000 (Vazyme, Nanjing, China) following the manufacturer’s protocol. Subsequently, cell supernatant and lysate were collected for Western blot analysis.

### 4.4. Formulation and Characterization of mRNA-LNPs

mRNA-LNP vaccines comprise RNA encapsulated in lipid nanoparticles (LNPs). The mRNA was prepared at 200 µg/mL in a 25 mM acetic acid-sodium acetate buffer (pH 5.5). LNPs were composed of ionizable cationic lipid, DSPC, cholesterol, and PEG-lipid in a 50:10:38.5:1.5 molar ratio. The mRNA-LNP formulation was synthesized using a microfluidic device (Micro & Nano, Shanghai, China) to combine aqueous and organic phases at a 3:1 flow ratio. Ethanol was replaced with a 25 mM Tris-HCl buffer (pH 7.5) using a 100 kDa ultrafiltration centrifugal filter to concentrate the solution, forming the mRNA-LNP vaccine. Encapsulation efficiency was evaluated with the Quant-iT^TM^ RiboGreen^TM^ RNA reagent (Thermo Fisher, Waltham, MA, USA), and particle size and zeta potential were measured using a Zetasizer Nano ZS (Malvern Panalytical, Malvern, UK).

### 4.5. Mouse Vaccination

In this study, female BALB/c mice (6–8 weeks old, SPF) were randomly assigned to two groups (n = 5). The experimental group received the mRNA-SP+P1 vaccine, while the control group was inoculated with the LNP formulation without mRNA molecules. The vaccine was administered intramuscularly at 20 μg per mouse, with booster doses on days 14 and 28. Serum samples were collected on days 14, 28, 42, 56, and 70 post-initial immunization. Splenocytes were harvested for flow cytometry analysis on day 42 following the primary vaccination.

### 4.6. ELISA for Specific IgG Antibody Titers

Serum IgG antibody titers against the *M. pneumoniae* P1 protein were quantified using an indirect antigen-specific enzyme-linked immunosorbent assay (ELISA). Microtiter plates were coated with 1 μg/mL of the P1 protein (YXX09301, Antibody system, Strasbourg, France) and incubated overnight at 4 °C. After blocking with 2% bovine serum albumin for 2 h at 37 °C, serial 2-fold dilutions of serum samples starting at 1:100 were added and incubated for 1 h at 37 °C. The plates were then washed and incubated with horseradish peroxidase (HRP)-conjugated goat anti-mouse IgG (K1221, APExBIO, Houston, TX, USA) for 1 h at 37 °C. Following additional washes, the colorimetric substrate 3,3′,5,5′-tetramethylbenzidine (TMB, SolarBio, Beijing, China) was added, and the reaction was stopped with 2M H_2_SO_4_ after 15 min of incubation in the dark. Absorbance was measured at 450 nm using a microplate reader (Bio-Tek Instruments, Winooski, VT, USA). ELISA endpoint titers were defined as the serum dilution yielding an absorbance at least 2.1 times that of the average negative serum (1:100).

### 4.7. Adhesion Inhibition

Mouse sera were collected 42 days post-primary immunization and inactivated at 56 °C for 30 min. KMB17 cells were seeded in a 6-well plate with cell slides and incubated overnight at 37 °C in a 5% CO_2_ atmosphere. A 500 μL aliquot of *M. pneumoniae* suspension (10^6^ CCU/mL) was combined with 100 μL of 10-fold diluted inactivated sera. After a 1-h pre-incubation at 37 °C, the mixture was added to the adherent KMB17 cell culture and incubated for 24 h at 37 °C in 5% CO_2_. The culture medium was removed, and each well was gently washed with PBS buffer (pH 7.4). Cells were fixed with 4% paraformaldehyde at room temperature for 10 min. Membrane permeabilization solution (50–100 μL) (G1204, Servicebio, Wuhan, China) was added to each well and incubated at room temperature for 20 min, followed by three PBS washes. The cell slides were blocked with 3% BSA at room temperature for 30 min. After removing the blocking solution, anti-*M. pneumoniae* antibody (ab53600, Abcam, Cambridge, UK) was added, and the slides were incubated overnight at 4 °C. The following day, slides were washed with PBS buffer (pH 7.4) and incubated with Cy3-labeled goat anti-rabbit IgG secondary antibody (GB21303, Servicebio, Wuhan, China) at room temperature for 50 min in the dark. After three additional PBS washes, DAPI staining solution was applied for 10 min in the dark. The slides were then mounted with an anti-fade medium, and images were captured using DAPI (excitation 330–380 nm/emission 420 nm) and Cy3 (excitation 510–560 nm/emission 590 nm) channels. Positivity was defined by specific fluorescent labelling of *M. pneumoniae* adhesion on the surface of KMB17 cells.

### 4.8. Flow Cytometry Assay

Spleen cells (1,000,000 cells/well) were stimulated with *M. pneumoniae* P1 protein (2 μg/mL) at 37 °C in 5% CO_2_ for 12 h. Brefeldin A (5 μg/mL, BioLegend, San Diego, CA, USA) was added for 4 h. Fc receptors were blocked using CD16/CD32 antibodies (Mouse BD Fc Block; Thermo Fisher, Waltham, MA, USA) for 15 min at 4 °C. Subsequently, cells were stained with fluorescently conjugated antibodies: CD4-FITC, CD8-FITC, CD44-PE, and CD62L-APC (Thermo Fisher, Waltham, MA, USA) for 30 min at 4 °C in the dark. After a final wash with cell staining buffer (BD Biosciences, San Jose, CA, USA), data were acquired using a FACS Calibur flow cytometer (BD Biosciences, San Jose, CA, USA) and analyzed with FlowJo software. The CD4^+^ Tem cell response was identified as live^+^/CD4^+^/CD44^+^/CD62L^−^, and the CD8^+^ Tem cell response was identified as live^+^/CD8^+^/CD44^+^/CD62L^−^.

### 4.9. RNA Sequencing

On day 42 post-immunization, whole blood samples were collected from mice for transcriptome analysis. Total RNA was extracted with Trizol reagent (Sangon, Shanghai, China), and its integrity was verified using an Agilent 2100 Bioanalyzer (Agilent Technologies, Santa Clara, CA, USA). The RNA was reverse transcribed with the Illumina NovaSeq kit (Illumina, San Diego, CA, USA) to create a cDNA library, followed by high-throughput sequencing on the Illumina NovaSeq 6000 platform (Illumina, San Diego, CA, USA). Raw sequencing data underwent quality assessment using FastQC (Version 0.11.2) and were trimmed using Trimmomatic (Version 0.36) for accuracy. Differential gene expression analysis was performed with DESeq2 (Version 1.12.4), and results were visualized. GO enrichment analysis utilized top GO (Version 2.24.0), while KEGG pathway enrichment analysis was conducted with clusterProfiler (Version 3.0.5).

### 4.10. Mouse Challenge

BALB/c mice received three immunizations with mRNA-SP+P1 (20 μg per mouse). To create an infection model, a 50-fold concentrated ATCC M129/ST3 drug-resistant bacterial solution (initially 10^7^ CCU/mL) was administered via nasal instillation (50 μL) on day 42/90 post initial immunization. Mice were monitored daily for body temperature and weight changes until euthanasia on the 4th day post challenge. Lung tissues were collected for *M. pneumoniae* load quantification via qPCR and histopathological evaluation using HE staining. Concurrently, whole blood samples were centrifuged to obtain serum, and the titers of specific IgG antibodies against P1 protein were detected using ELISA.

### 4.11. Histopathology

Lung tissue was fixed in the fixative (Servicebio, Wuhan, China) for 48 h, followed by paraffin embedding. Sections 5 μm thick were stained with hematoxylin and eosin (HE) and digitally scanned using the 3DHISTECH system. An experienced pathologist evaluated the HE-stained sections with Case Viewer software. Scoring criteria included: A. percentage of peribronchiolar infiltration; B. severity of peribronchiolar infiltration; C. severity of luminal exudation; D. percentage of perivascular infiltration; and E. severity of interstitial pneumonia. The total pathology score was calculated as A + 3(B + C) + D + E, providing a comprehensive assessment of lung damage in mice.

### 4.12. Quantification of Mycoplasma pneumoniae Load

Lung tissue was weighed and homogenized, and genomic DNA was extracted using the TIANamp Genomic DNA Kit (TIANGEN, Beijing, China). Quantitative detection was conducted with the CFX Connect Real-time PCR System (Bio-Rad, Hercules, CA, USA). Primers and probes were specifically designed for the *M. pneumoniae* 16S rRNA gene: 16S rRNA -F: TAACGGCCTACCAAGGCAATGA;16SrRNA-R: AGTCAAACTCTAGCCATTACCTGC;Probe-16SrRNA: ACGCCCATACTCCTACGGGAGGCAGCAGT. A standard curve was generated using plasmids containing the 16S rRNA gene fragment, and qRT-PCR was performed with the AceQ Universal U^+^ Probe Master Mix V2 (Vazyme, Nanjing, China) following the manufacturer’s protocol.

### 4.13. Statistical Analysis

Statistical analyses were conducted using GraphPad Prism 10.0 (GraphPad Software). Data are expressed as mean ± SD. Differences were assessed using unpaired *t*-tests or Mann-Whitney tests or two-way ANOVA. A *p*-value of <0.05 was considered statistically significant. Significance levels are indicated by * *p* < 0.05, ** *p* < 0.01, *** *p* < 0.001, and **** *p* < 0.0001.

## 5. Conclusions

This study assessed an mRNA vaccine targeting the P1 protein of *M. pneumoniae*, specifically its C-terminal region. In BALB/c mice, the vaccine elicited a humoral immune response and an effector memory T cell response. Importantly, it conferred significant and long-lasting, though partial, protection against the ATCC M129 strain and partial cross-protection against the drug-resistant ST3 strain. These findings indicate that the mRNA-SP+P1 vaccine offers a novel strategy for preventing *M. pneumoniae* infection and serves as a promising model for the development of vaccines targeting other drug-resistant pathogens.

## Figures and Tables

**Table 1:** Drug resistance results of an ST3 drug-resistant strain obtained by whole-genome sequencing.

CARD	ARO Name	Gene Family	Drug Class
ARO: 3000024	patA	ATP-binding cassette (ABC) antibiotic efflux pump	fluoroquinolone antibiotic
ARO: 3000191	tet(Q)	tetracycline resistant ribosomal protection protein	tetracycline antibiotic
ARO: 3000501	rpoB2	rifamycin resistant beta subunit of RNA polymerase (rpoB)	rifamycin antibiotic
ARO: 3000510	Staphylococcus aureus mupB confening resistance to mupirocin	antibiotic resistant isoleucyl tRNA synthetase (ileS)	mupirocin-like antibiotic
ARO: 3000521	Staphylococcus aureus mupA confenring resistance to mupirocin	antibiotic resistant isoleucyl tRNA synthetase (ileS)	mupirocin-like antibiotic
ARO: 3000535	macB	ATP-binding cassette (ABC) antibiotic efflux pump	macrolide antibiotic
ARO: 3002882	lmrD	ATP-binding cassette (ABC) antibiotic efflux pump	lincosamide antibiotic
ARO: 3003748	oleC	ATP-binding cassette (ABC) antibiotic efflux pump	macrolide antibiotic
ARO: 3003948	efrA	ATP-binding cassette (ABC) antibiotic efflux pump	Fluoroquinolone antibiotic; macrolide antibiotic; rifamycin antibiotic
ARO: 3003949	efrB	ATP-binding cassette (ABC) antibiotic efflux pump	Fluoroquinolone antibiotic; macrolide antibiotic; rifamycin antibiotic
ARO: 3003950	msbA	ATP-binding cassette (ABC) antibiotic efflux pump	nitroimidazole antibiotic
ARO: 3004480	Bifidobacterium adolescentis rpoB mutants conferring resistance to rifampicin	rifamycin resistant beta subunit of RNA polymerase (rpoB)	rifamycin antibiotic

CARD: ARO number of The Comprehensive Antibiotic Resistance Database; ARO name: resistance gene corresponding to ARO number; Gene Family: resistance gene family; Drug Class: antibiotic type.

## Data Availability

All relevant data are within the paper.
